# Association of anthropometric and biochemical–anthropometric obesity indices with chronic kidney disease in diabetes: a KNHANES-based study

**DOI:** 10.1007/s10157-026-02833-w

**Published:** 2026-02-26

**Authors:** Eun Mi Yang, Jong Im Won, Sang Heon Suh, Hong Sang Choi, Chang Seong Kim, Eun Hui Bae, Seong Kwon Ma, Soo Wan Kim

**Affiliations:** 1 Departments of Pediatrics, Chonnam National University Medical School, Chonnam National University Hospital, Gwangju, South Korea; 2 Department of Internal Medicine, Chonnam National University Medical School, Gwangju, South Korea; 3 Departments of Internal Medicine, Chonnam National University Medical School, Chonnam National University Hospital, 42 Jebongro, Gwangju, 61469 South Korea

**Keywords:** Diabetes mellitus, Chronic kidney disease, Obesity-related indices, Sex, Anthropometric measurement

## Abstract

**Background:**

Obesity is a known risk factor for diabetes mellitus (DM) and chronic kidney disease (CKD), yet the relationship between various obesity-related indices and CKD remains unclear. This study evaluated the associations between eight obesity indices with CKD and identified the most useful index among patients with DM.

**Methods:**

This study used data from the Korean National Health and Nutrition Examination Survey conducted from 2007 to 2018. A total of 5,067 participants aged ≥ 20 years with DM were included. The study evaluated four traditional anthropometric obesity indices (body mass index [BMI], waist-to-height ratio [WHtR], body roundness index [BRI], conicity index [CI]) and four biochemical-anthropometric indices, including two Asian-specific indices (lipid accumulation product [LAP], visceral adiposity index [VAI], Chinese visceral adiposity index [CVAI], and new visceral adiposity index [NVAI]).

**Results:**

WHtR, BRI, CI, CVAI, and NVAI were higher in males with CKD, while only CI, CVAI, and NVAI were elevated in females. All eight indices were independently associated with CKD risk in males, whereas only the anthropometric-biochemical indices LAP, VAI, and CVAI were significantly associated with CKD in females. NVAI in males and CVAI in females exhibited the highest area under the curve values of 0.615 and 0.658, respectively.

**Conclusions:**

Various obesity indices were associated with CKD in patients with DM, although the associations differed by sex. Asian-specific indices may be the most useful for reflecting CKD in patients with DM.

## Introduction

Diabetes mellitus (DM) is a major global health burden, affecting about 10.5% of adults aged 20–79 years in 2021, and projected to reach 12.2% by 2045 [1, 2]. It contributes substantially to global mortality and morbidity across ages and regions [3]. Chronic kidney disease (CKD), another escalating global health concern, commonly coexists with DM. Nearly one in three adults with DM has CKD, while up to 40% of individuals with DM are likely to develop CKD during their lifetime [4]. Their coexistence markedly increases mortality, disease complexity, and healthcare costs [5]. The global obesity epidemic has further increased type 2 DM and diabetes-related kidney disease, a growing cause of end-stage kidney disease (ESKD).

Obesity is a major risk factor for both DM development and CKD progression. Although the body mass index (BMI) is the most widely used clinical indicator for identifying obesity [6], it does not accurately reflect visceral fat distribution [7]. Increasing evidence suggests that not just the amount, but the distribution of visceral adipose tissue, plays a critical role in DM-associated metabolic complications [8], contributing to kidney injury directly and through comorbidities such as hypertension, and hyperlipidemia [9]. Thus, anthropometric obesity indices based on waist circumference (WC) are used to more accurately assess visceral obesity. Additionally, biochemical–anthropometric obesity indices that incorporate lipid profiles are more representative of visceral adipose tissue [10, 11]. However, these indices have been largely validated in predominantly Caucasian populations; therefore, they do not account for the unique clinical characteristics of Asians, who accumulate more visceral fat despite having a relatively low BMI [12]. Consequently, Asian-specific indices have been developed and applied to more accurately reflect these ethnic differences in body fat distribution [12–14].

This study aimed to examine the association between various obesity-related indices and CKD in patients with DM using nationally representative data to provide deeper insights into the risk stratification for CKD in this high-risk population.

## Materials and methods

### Study population and data collection

**Fig. 1:**
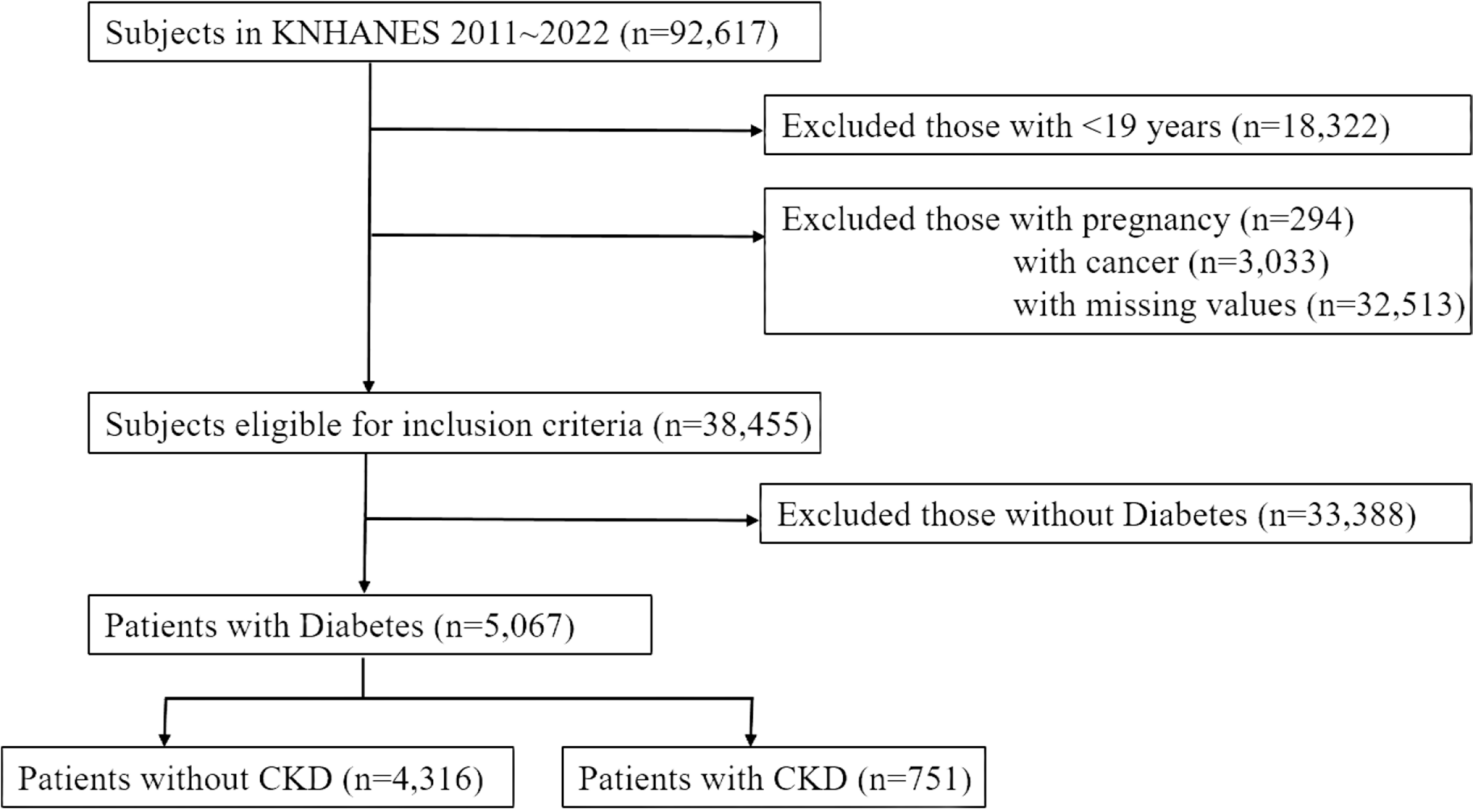
Flowchart of participant recruitment for the study. KNHANES, Korea National Health and Nutrition Examination Survey; CKD, chronic kidney disease

This study used data from the Korea National Health and Nutrition Examination Survey (KNHANES) phases IV–VII (2007–2018), a nationally representative cross-sectional survey by the Division of Chronic Disease Surveillance at the Korea Centers for Disease Control and Prevention. Of 92,617 participants aged ≥ 19 years included in the dataset, we excluded participants who were pregnant, had a history of cancer, or had missing values. Of these, 5,067 participants with DM were included in the final analysis (Fig. 1). Data on anthropometric, biochemical, socioeconomic, lifestyle, and medical factors were collected via standardized questionnaires and measurements by trained professionals. Demographic data (age, sex, income, education, alcohol use, and DM history) were self-reported. Laboratory tests included fasting glucose, glycated hemoglobin (HbA1c), total cholesterol, triglyceride (TG), high-density lipoprotein (HDL) cholesterol, blood urea nitrogen, and creatinine were measured in the morning after fasting for at least 8 h. Proteinuria was assessed by dipstick, and estimated glomerular filtration rate (eGFR) was calculated using the CKD-EPI equation [15]. Blood pressure (BP) was measured three times at 30 s intervals after 5 min of rest, using the mean of the second and third readings.

### Definition of each criterion and variable

DM was defined as a self-reported physician-diagnosed or survey-identified condition with fasting glucose ≥ 126 mg/dL and/or HbA1c ≥ 6.5%. CKD was defined as eGFR ≤ 60 mL/min/1.73 m^2^ or spot proteinuria ≥ 1 +. Household income was classified into quartiles (low, lower-middle, upper-middle, high) based on equivalized income. Heavy alcohol drinking was ≥ 7 drinks per occasion for men and ≥ 5 for women at least once per month. Current smoking indicated smoking at the time of survey. Daytime workers were those reporting primary work during standard daytime hours.

Calculation of obesity-related indices.

1. BMI was calculated as BMI = weight (kg)/height^2^ (m).

2. Waist-to-height ratio (WHtR) was calculated as WHtR = WC (cm)/height (cm).

3. Body roundness index (BRI) was calculated as: BRI = 364.2 ‒ 365.5 x $\sqrt{1-(\frac{\text{WC }\left(\mathrm{m}\right) /2\uppi }{0.5\text{ x body height }\left(\mathrm{m}\right)}}$).^2^ [16].

4. Conicity index (CI) was calculated as $CI=\frac{\mathrm{WC}(\mathrm{m})}{0.109\text{ X }\sqrt{\frac{\text{body weight }(\mathrm{kg})}{\text{body height }(\mathrm{m})}}}$ [17].

5. Lipid accumulation product (LAP) was calculated for man as LAP = (WC (cm) ‒ 65) x TG.

and for woman as: LAP = (WC (cm) ‒ 58) x TG [10].

6. Visceral adiposity index (VAI) score was calculated for man as VAI = $\left(\frac{\text{WC }(\mathrm{cm})}{39.68+(1.88\text{ x BMI})}\right)\times \left(\frac{\mathrm{TG}}{1.03}\right)\times \left(\frac{1.31}{\mathrm{HDL}}\right)$.

And for woman as VAI = $\left(\frac{\text{WC }(\mathrm{cm})}{36.58+(1.89\text{ x BMI})}\right)\times \left(\frac{\mathrm{TG}}{0.81}\right)\times \left(\frac{1.52}{\mathrm{HDL}}\right)$ [11].

7. Chinese visceral adiposity index (CVAI) was calculated for man as: CVAI = –267.93 + (0.68 × age) + (0.03 × BMI) + (4.00 × WC) + (22 × logTG) – (16.32 × HDL).

And for woman as CVAI = –187.32 + (1.71 × age) + (4.23 × BMI) + (1.12 × WC) + (39.76 × logTG) – (11.66 × HDL) [13].

8. New visceral adiposity index (NVAI) was calculated for man as

$$NVAI=\frac{1}{[1+{e}^{-(-21.858 + \left(0.099 \times \text{ age}\right) + \left(0.10 \times \text{ WC}\right) + \left(0.12 \times \text{ mean BP}\right) + \left(0.006 \times \text{ TG}\right) + \left(-0.077 \times \text{ HDL}\right)}]}$$

and for woman as

$$NVAI=\frac{1}{[1+{e}^{-(-18.765 + \left(0.058 \times \text{ age}\right) + \left(0.14 \times \text{ WC}\right) + \left(0.057 \times \text{ mean BP}\right) + \left(0.004 \times \text{ TG}\right) + \left(-0.057 \times \text{ HDL}\right)}]}$$ [14].

Since hip circumference data were not available in the KNHANES dataset, indices requiring hip circumference could not be assessed.

### Statistical analyses

Sampling and weighting were performed according to a stratified survey design using appropriate sample weights. Nominal variables were presented as frequencies and percentages, and continuous variables as means ± SD. Differences between DM patients with and without CKD were assessed using the Student’s *t*-test and the Chi-square test. Multivariable logistic regression was used to identify factors associated with CKD, adjusting for age, sex, income, education, alcohol consumption, and work schedule; smoking status was additionally adjusted for in males. Receiver operating characteristic (ROC) curves and the corresponding area under the curve (AUC) values were used to evaluate the predictive performance of obesity-related indices for CKD. Statistical significance was set at *P* ≤ 0.05. All analyses were performed using STATA version 16.4 (StataCorp, College Station, TX, USA).

## Results

### Comparison of baseline characteristics according to the CKD status

Among 5,067 patients (mean age 58.2 ± 12.4 years), 12.0% had CKD. The cohort comprised 2,643 males and 2,597 females, with CKD prevalence of 13.7% and 9.9%, respectively. Table 1 shows baseline characteristics by CKD status. Compared to those without CKD, patients with CKD were older, predominantly male, less educated, less likely to be daytime workers or heavy drinkers, and more likely to have a lower income. Height and weight did not differ, whereas WC was significantly larger in DM patients and CKD. Furthermore, CKD patients had higher systolic BP and HbA1c levels but lower diastolic BP, total cholesterol, and HDL-cholesterol levels. Among the obesity-related indices, WHtR, BRI, CI, CVAI and NVAI were significantly higher in CKD patients, while BMI showed no difference. LAP and VAI were higher, although without statistical significance (Table 2).

**Table 1:** Comparison of baseline characteristics between patients with DM according to the CKD status

Characteristics	Non-CKD (N = 4,316)	CKD (N = 751)	*P* value
Age (years)	58.21 ± 12.42	66.28 ± 11.88	< 0.001
Male (%)	55.5	64.3	0.002
Urban residence (%)	78.0	77.3	0.763
Household income (%)			< 0.001
Low	25.4	44.5	
Middle-low	26.9	25.8	
Middle-high	24.3	14.7	
High	23.4	15.0	
Education level (%)			< 0.001
≤ Primary school	33.7	45.6	
Middle school	16.7	11.9	
High school	30.0	27.5	
≥ College	19.6	15.0	
Heavy alcohol drinking (%)	34.5	20.8	< 0.001
Smoking (%)	25.7	23.0	0.302
Daytime workers (%)	56.3	39.3	< 0.001
Body height (cm)	162.65 ± 9.67	161.39 ± 9.38	0.434
Body weight (kg)	67.83 ± 13.11	66.63 ± 12.75	0.290
Waist circumference (cm)	88.08 ± 9.36	89.57 ± 9.59	0.027
Systolic BP (mmHg)	125.46 ± 16.42	130.68 ± 19.64	< 0.001
Diastolic BP (mmHg)	77.03 ± 10.60	73.59 ± 13.53	< 0.001
Laboratory parameters			
Fasting glucose (mg/dL)	141.12 ± 40.99	139.13 ± 43.65	0.594
HbA_1C_ (%)	7.23 ± 1.37	7.39 ± 1.38	0.024
Total cholesterol (mg/dL)	187.43 ± 43.69	179.33 ± 43.54	0.002
Triglyceride (mg/dL)	184.70 ± 157.14	195.88 ± 170.36	0.329
HDL-cholesterol (mg/dL)	45.82 ± 10.97	42.91 ± 10.49	< 0.001
Blood urea nitrogen (mg/dL)	15.33 ± 4.15	20.99 ± 8.65	< 0.001
Creatinine (mg/dL)	0.83 ± 0.17	1.27 ± 0.72	< 0.001
eGFR (mL/min/1.73m^2^)	90.51 ± 13.75	60.99 ± 22.81	< 0.001

CKD, chronic kidney disease; DM, diabetes mellitus; BP, blood pressure; HbA_1C_, glycated hemoglobin; eGFR, estimated glomerular filtration rate

Values are presented as number (%) or mean (SD)

**Table 2:** Obesity-related indices in patients with DM with or without CKD

	Non-CKD (*N* = 4,316)	CKD (*N* = 751)	*P* value
BMI (kg/m^2^)	25.53 ± 3.69	25.44 ± 3.62	0.332
WHtR (%)	54.26 ± 5.89	55.55 ± 5.97	0.022
BRI	4.26 ± 1.25	4.53 ± 1.29	0.036
CI	1.26 ± 0.07	1.28 ± 0.07	< 0.001
LAP	55.87 ± 53.30	61.45 ± 59.35	0.377
VAI	3.01 ± 3.33	3.47 ± 4.67	0.146
CVAI	124.54 ± 35.66	138.28 ± 35.42	< 0.001
NVAI	0.71 ± 0.30	0.83 ± 0.24	< 0.001

CKD, chronic kidney disease; DM, diabetes mellitus; BMI, body mass index, WHtR, waist-to-height ratio; BRI, body roundness index; CI, conicity index, LAP, lipid adiposity index; VAI, visceral adiposity index; CVAI, Chinese visceral adiposity index; NVAI, new visceral adiposity index

Values are presented as mean (SD)

### Sex-specific differences in baseline characteristics by the CKD status

When stratified by sex, several baseline characteristics differed from those observed in the overall group. In male, height was significantly lower and current smoking was less prevalent in patients with CKD, whereas WC and total cholesterol showed no significant differences. In females, both height and weight were significantly lower in the CKD group, while WC and HbA1c did not differ significantly between the groups (Table 3). Regarding obesity indices, males showed patterns similar to the total cohort: WHtR, BRI, CI, CVAI, and NVAI were significantly higher in the CKD group, whereas LAP and VAI were higher but not statistically significant. In females, CI, CVAI, and NVAI were higher in the CKD group, while other indices showed no significant differences (Table 4).

**Table 3:** Comparison of baseline characteristics according to the CKD status and sex in patients with DM

	Male			Female		
Characteristics	Non-CKD (*N* = 2,142)	CKD (*N* = 434)	*P* value	Non-CKD (*N* = 2,174)	CKD (*N* = 317)	*P* value
Age (years)	55.72 ± 11.83	63.91 ± 11.93	<.001	61.34 ± 12.43	69.88 ± 10.88	< 0.001
Urban residence (%)	78.3	77.3	0.765	77.7	77.2	0.894
Household income (%)			<.001			< 0.001
Low	19.2	39.1		33.3	54.4	
Middle-low	25.6	28.1		28.5	21.8	
Middle-high	25.9	16.3		22.2	11.6	
High	29.3	16.5		16.1	12.2	
Education level (%)			<.001			< 0.001
≤ Middle school	37.2	44.3		50.7	70.1	
High school	35.2	34.5		23.6	14.7	
≥ College	27.6	21.2		9.7	3.7	
Heavy alcohol drinking (%)	53.9	31.1	<.001	10.3	2.2	0.004
Smoking (%)	42.2	33.6	0.028	5.1	4.0	0.456
Daytime workers (%)	68.9	47.4	0.001	40.6	24.6	0.021
Body height (cm)	169.17 ± 6.34	167.37 ± 6.02	<.001	154.44 ± 6.31	152.36 ± 5.54	0.006
Body weight (kg)	72.73 ± 12.45	71.24 ± 12.43	0.074	61.68 ± 11.17	59.64 ± 9.70	0.042
Waist circumference (cm)	89.37 ± 8.93	90.71 ± 9.38	0.084	86.45 ± 9.64	87.86 ± 9.67	0.496
Systolic BP (mmHg)	124.69 ± 15.45	130.79 ± 18.71	< 0.001	126.43 ± 17.52	130.50 ± 21.01	0.071
Diastolic BP (mmHg)	78.88 ± 10.70	75.72 ± 13.68	< 0.001	74.71 ± 10.00	70.36 ± 12.66	< 0.001
Laboratory parameters						
Fasting glucose (mg/dL)	144.42 ± 40.58	140.79 ± 42.48	0.462	136.96 ± 41.14	136.60 ± 45.31	0.807
HbA_1C_ (%)	7.26 ± 1.41	7.45 ± 1.46	0.028	7.20 ± 1.31	7.29 ± 1.25	0.574
Total cholesterol (mg/dL)	184.76 ± 43.33	179.79 ± 44.85	0.248	190.79 ± 43.91	178.63 ± 41.56	0.002
Triglyceride (mg/dL)	204.31 ± 174.99	205.20 ± 180.15	0.684	160.02 ± 127.07	181.76 ± 153.58	0.401
HDL-cholesterol (mg/dL)	44.22 ± 10.64	41.93 ± 10.31	0.004	47.84 ± 11.05	44.39 ± 10.59	< 0.001
BUN (mg/dL)	15.51 ± 4.11	20.63 ± 8.10	< 0.001	15.11 ± 4.19	21.55 ± 9.41	< 0.001
Creatinine (mg/dL)	0.93 ± 0.13	1.34 ± 0.72	< 0.001	0.71 ± 0.11	1.17 ± 0.71	< 0.001
eGFR (mL/min/1.73m^2^)	90.70 ± 13.49	63.95 ± 23.01	< 0.001	90.27 ± 14.08	56.49 ± 21.76	< 0.001

CKD, chronic kidney disease; DM, diabetes mellitus; BP, blood pressure; HbA_1C_, glycated hemoglobin; eGFR, estimated glomerular filtration rate

Values are presented as number (%) or mean (SD)

**Table 4:** Obesity-related indices in patients with DM, stratified by sex and CKD status

Obesity-related indices	Male			Female		
	Non-CKD	CKD	*P* value	Non-CKD	CKD	*P* value
BMI	25.33 ± 3.48	25.31 ± 3.59	0.719	25.79 ± 3.92	25.65 ± 3.66	0.442
WHtR	52.84 ± 5.08	54.11 ± 5.26	0.003	56.04 ± 6.35	57.70 ± 6.33	0.120
BRI	3.96 ± 1.03	4.21 ± 1.10	0.005	4.64 ± 1.38	5.00 ± 1.40	0.143
CI	1.25 ± 0.06	1.28 ± 0.06	<.001	1.26 ± 0.08	1.29 ± 0.07	0.004
LAP	58.39 ± 59.19	60.84 ± 56.70	0.576	52.70 ± 44.62	62.38 ± 63.22	0.462
VAI	2.95 ± 3.24	3.06 ± 2.84	0.449	3.10 ± 3.44	4.08 ± 6.47	0.089
CVAI	125.58 ± 37.29	136.16 ± 36.91	0.001	123.23 ± 33.45	141.45 ± 32.88	< 0.001
NVAI	0.82 ± 0.25	0.90 ± 0.18	<.001	0.59 ± 0.31	0.72 ± 0.28	< 0.001

CKD, chronic kidney disease; DM, diabetes mellitus; BMI, body mass index; WHtR, waist-to-height ratio; BRI, body roundness index; CI, conicity index; LAP, lipid adiposity index; VAI, visceral adiposity index; CVAI, Chinese visceral adiposity index; NVAI, new visceral adiposity index

Values are presented as mean (SD)

### Association between obesity-related indices and CKD

Logistic regression analyses were performed in patients with DM to assess the association between obesity-related indices and CKD (Table 5). In the univariate analysis, most obesity-related indices (except for BMI, LAP, and VAI) were significantly associated with CKD. After adjusting for age and sex, the significant associations persisted across all indices. Even after adjusting for multiple variables, all obesity-related indices were associated with an increased risk of CKD: BMI (odds ratio [OR] = 1.053; *P* = 0.009), WHtR (OR = 1.031; *P* = 0.007), BRI (OR = 1.150; *P* = 0.009), CI (OR = 1.229; *P* = 0.027), LAP (OR = 1.004; *P* < 0.001), VAI (OR = 1.056; *P* = 0.002), CVAI (OR = 1.008; *P* < 0.001) and NVAI (OR = 2.866; *P* = 0.001).

**Table 5:** Association between the obesity-related indexes and CKD in patients with DM in multivariable logistic regression analysis

	Model 1^a^			Model 2^b^			Model 3^c^		
	OR	95% CI	*P* value	OR	95% CI	*P* value	OR	95% CI	*P* value
BMI (per 1 kg/m^2^)	0.984	0.952–1.017	0.342	1.043	1.003–1.086	0.036	1.053	1.013–1.094	0.009
WHtR	1.022	1.003–1.041	0.020	1.026	1.003–1.049	0.026	1.031	1.008–1.055	0.007
BRI (per 1)	1.099	1.009–1.197	0.030	1.123	1.011–1.249	0.031	1.150	1.035–1.278	0.009
CI (per 0.1)	1.562	1.333–1.830	<.001	1.207	1.015–1.435	0.034	1.229	1.023–1.475	0.027
LAP (per 1)	1.001	0.999–1.003	0.352	1.004	1.002–1.006	0.001	1.004	1.002–1.007	< 0.001
VAI (per 1)	1.021	0.989–1.053	0.197	1.053	1.015–1.093	0.006	1.056	1.020–1.094	0.002
CVAI (per 1)^d^	1.010	1.006–1.013	<.001	1.010	1.006–1.013	< 0.001	1.008	1.004–1.012	< 0.001
NVAI (per 1)^d^	4.428	2.554–7.676	<.001	4.237	2.337–7.684	< 0.001	2.866	1.529–5.371	0.001

CKD, chronic kidney disease; DM, diabetes mellitus; BMI, body mass index; WHtR, waist-to-height ratio; BRI, body roundness index; CI, conicity index; LAP, lipid adiposity index; VAI, visceral adiposity index; CVAI, Chinese visceral adiposity index; NVAI, new visceral adiposity index; OR, odds ratio; CI, confidence interval

^a^Univariate

^b^Adjusted for age and sex

^c^Multivariable model adjusted for age, household income, education level, alcohol consumption, and work schedule pattern

^d^Adjusted for variables except age

Sex-stratified analyses revealed consistent results in males, with all indices showing significant associations with CKD in patients with DM: BMI (OR = 1.077; *P* = 0.012), WHtR (OR = 1.049; *P* = 0.007), BRI (OR = 1.261; *P* = 0.007), CI (OR = 1.361; *P* = 0.024), LAP (OR = 1.004; *P* = 0.004), VAI (OR = 1.079; *P* = 0.007), CVAI (OR = 1.007; *P* = 0.001) and NVAI (OR = 3.773; *P* = 0.013). In females, only LAP (OR = 1.004; *P* = 0.012), VAI (OR = 1.052; *P* = 0.008), and CVAI (OR = 1.010; *P* = 0.002) remained significantly associated with CKD in patients with DM (Table 6).

**Table 6:** Association between the obesity-related indexes and CKD in patients with DM in multivariable logistic regression analysis according to sex

	Model 1^a^			Model 2^b^			Model 3^c^		
Male	OR	95% CI	*P* value	OR	95% CI	*P* value	OR	95% CI	*P* value
BMI (per 1 kg/m^2^)	0.991	0.943–1.041	0.722	1.063	1.002–1.129	0.043	1.077	1.016–1.141	0.012
WHtR	1.047	1.017–1.079	0.002	1.042	1.007–1.078	0.019	1.049	1.014–1.086	0.007
BRI (per 1)	1.246	1.079–1.439	0.003	1.219	1.029–1.443	0.022	1.261	1.065–1.493	0.007
CI (per 0.1)	1.847	1.441–2.367	< 0.001	1.339	1.040–1.723	0.023	1.361	1.041–1.780	0.024
LAP (per 1)	1.001	0.998–1.004	0.556	1.004	1.001–1.008	0.005	1.004	1.001–1.008	0.004
VAI (per 1)	1.021	0.971–1.074	0.419	1.076	1.020–1.136	0.008	1.079	1.021–1.140	0.007
CVAI (per 1)^d^	1.007	1.003–1.012	< 0.001				1.007	1.003–1.012	0.001
NVAI (per 1)^d^	5.573	2.011–15.443	0.001				3.773	1.324–10.755	0.013
Female	OR	95% CI	*P* value	OR	95% CI	*P* value	OR	95% CI	*P* value
BMI (per 1 kg/m^2^)	0.983	0.941–1.027	0.452	1.020	0.969–1.074	0.450	1.031	0.981–1.084	0.230
WHtR	1.021	0.995–1.048	0.114	1.009	0.980–1.039	0.545	1.015	0.986–1.046	0.311
BRI (per 1)	1.094	0.974–1.229	0.130	1.044	0.912–1.194	0.534	1.073	0.939–1.225	0.311
CI (per 0.1)	1.380	1.108–1.720	0.004	1.070	0.837–1.367	0.590	1.112	0.856–1.444	0.427
LAP (per 1)	1.001	0.998–1.004	0.479	1.003	1.000–1.007	0.057	1.004	1.001–1.008	0.012
VAI (per 1)	1.024	0.984–1.065	0.238	1.041	1.001–1.082	0.044	1.052	1.013–1.092	0.008
CVAI (per 1)^d^	1.014	1.008–1.020	< 0.001				1.010	1.004–1.017	0.002
NVAI (per 1)^d^	3.437	1.655–7.137	0.001				2.030	0.942–4.373	0.070

CKD, chronic kidney disease; DM, diabetes mellitus; BMI, body mass index; WHtR, waist-to-height ratio; BRI, body roundness index; CI, conicity index; LAP, lipid adiposity index; VAI, visceral adiposity index; CVAI, Chinese visceral adiposity index; NVAI, new visceral adiposity index; OR, odds ratio; CI, confidence interval

^a^Univariate

^b^Adjusted for age

^c^Multivariable model adjusted for age, household income, education level, alcohol consumption, and work schedule pattern in females; in males, smoking was additionally included as a covariate

^d^Adjusted for variables except age

### Discriminative performance of obesity-related indices for predicting CKD

**Fig. 2:**
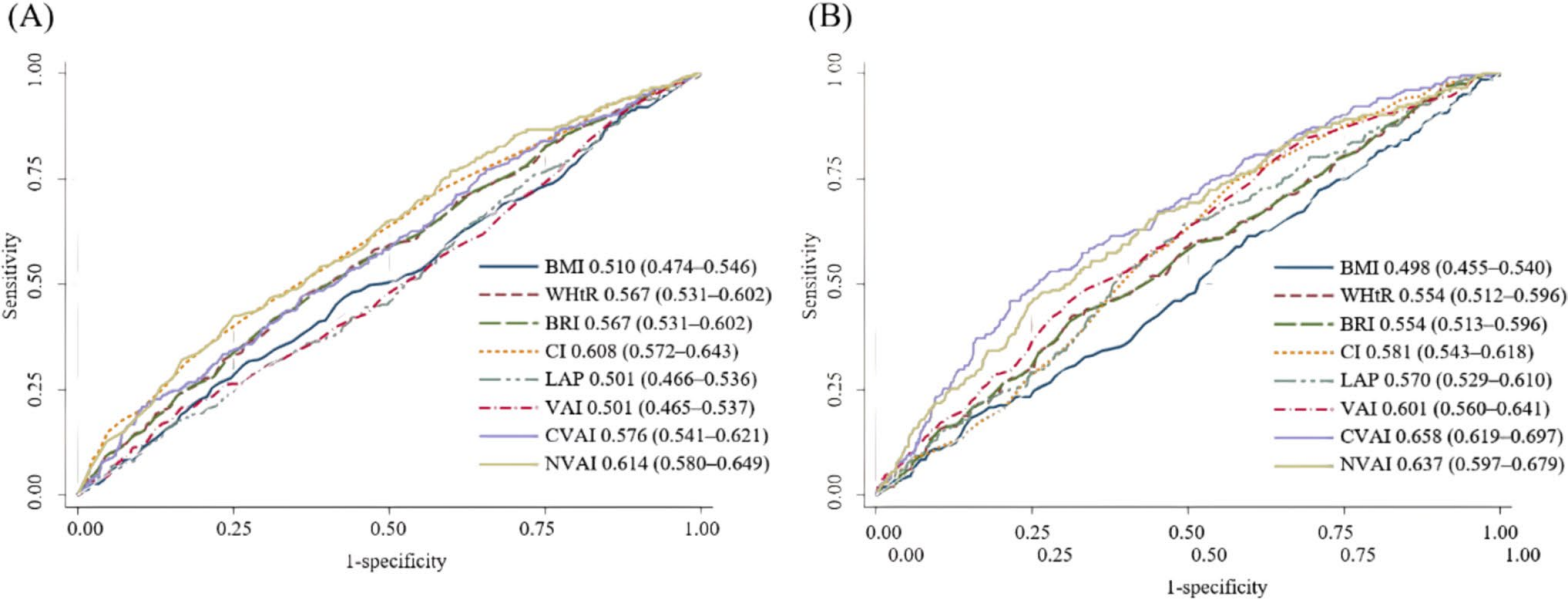
Comparison of the predictive performance of eight obesity-related parameters for the diagnosis of chronic kidney disease in patients with diabetes mellitus. **A** Males, **B** Females. Predictive values were calculated per 1 kg/m^2^ increase in BMI, per 0.1-unit increase in CI, and per 1-unit increase in all other parameters. Data in the legend represent the area under the curve followed by the 95% confidence interval in parentheses. BMI, body mass index; WHtR, waiist-to-height ratio; BRI, body roundness index; CI, conicity index; LAP, lipid adiposity index; VAI, visceral adiposity index; CVAI, Chinese visceral adiposity index; NVAI, new visceral adiposity index

The predictive performance of obesity-related indices for CKD patients with DM was assessed using ROC curves and AUCs (Table 7). Among the obesity indices, NVAI demonstrated the highest predictive ability (AUC = 0.636), followed by CVAI (AUC 0.608), CI (AUC = 0.594), WHtR (AUC = 0.542), BRI (AUC = 0.542), VAI (AUC = 0.532), and BMI (AUC = 0.510). In sex-stratified analysis, NVAI (AUC = 0.615) showed the statistically significant discriminative ability in males, followed by CI (AUC = 0.608), CVAI (AUC = 0.576), BRI (AUC = 0.567) and WHtR (AUC = 0.567), BMI (AUC = 0.510), and LAP (AUC = 0.501) and VAI (AUC = 0.501) (Fig. 2). In females, CVAI (AUC = 0.658) showed the statistically significant discriminative ability, followed by NVAI (AUC = 0.637), VAI (AUC = 0.601), CI (AUC = 0.581), LAP (AUC = 0.570), WHtR (AUC = 0.554), and BRI (AUC = 0.554), while BMI showed the lowest discriminative ability (AUC = 0.498).

**Table 7:** Association between the obesity-related indexes and CKD in patients with DM

Obesity-related indices	Overall	
	AUC	95% CI
BMI (per 1 kg/m^2^)	0.510	0.483–0.537
WHtR (per 1)	0.542	0.515–0.569
BRI (per 1)	0.542	0.513–0.569
CI (per 0.1)	0.594	0.569–0.630
LAP (per 1)	0.525	0.498–0.552
VAI (per 1)	0.532	0.505–0.560
CVAI (per 1)	0.608	0.521–0.634
NVAI (per 1)	0.636	0.610–0.662

CKD, chronic kidney disease; DM, diabetes mellitus; BMI, body mass index; WHtR, waist-to-height ratio; BRI, body roundness index; CI, conicity index; LAP, lipid adiposity index; VAI, visceral adiposity index; CVAI, Chinese visceral adiposity index; NVAI, new visceral adiposity index; AUC, area under the curve; CI, confidence interval

## Discussion

This study evaluated various obesity-related anthropometric indices to determine their associations with CKD risk in patients with DM. Eight indices showed significant associations with increased CKD risk, indicating that obesity plays a role in CKD of patients affected by DM. In males, all indices were positively associated with CKD risk, with NVAI showing the strongest association. In contrast, anthropometric based indices were not significant in females, while only biochemical–anthropometric indices increased CKD risk.

Obesity is fundamentally linked to a spectrum of metabolic comorbidities, including type 2 DM, malignancy, hypertension, dyslipidemia, and cardiovascular disease [18]. Beyond these association, obesity serves as a critical driver of CKD development and progression, exerting delicious effects independent of DM status [19]. Pathophysiologically, adipocytes function as active endocrine units that secrete various adipokines; these molecules exert direct nephrotoxic [20] and indirectly impair kidney function by promoting insulin resistance [21]. Conversely, the presence of CKD can precipitate reciprocal endocrine and immunologic dysregulations within adipose tissue, creating a maladaptive feedback loop [22]. These clinical implications are particularly salient in individuals with DM, who face more than a twofold higher risk of developing CKD compared to the non-diabetic population [23]. This risk further compounded by concomitant obesity [24, 25], which exacerbates kidney injury through complex synergistic mechanisms. In addition to hyperglycemia-induced fibrosis and renin–angiotensin–aldosterone system-medicated hemodynamic alteration, visceral adiposity triggers chronic systemic inflammation [22, 26]. This persistent inflammatory milieu accelerates structural pathologies, such as mesangial expansion on and glomerular hypertrophy, ultimately culminating podocyte loss and irreversible kidney damage [21]. Consequently, this study aimed to elucidate the association between various obesity-related indices and the risk of CKD specifically with the diabetic population to refine risk stratification in this high-risk group.

Various obesity-related indices are used to evaluate obesity, including BMI, WHtR, BRI [16], CI [17], LAP [10], and VAI [11]. Although BMI is a widely used traditional index, it has inherent limitations, particularly its inability to distinguish between lean body mass and fat mass. Therefore, indices that combine anthropometric and biochemical measurements, such as LAP, VAI, CVAI and NVAI, have recently been proposed as surrogate markers of visceral adiposity. Furthermore, Asian-specific indices, including CVAI and NVAI, have been developed to reflect ethnic differences in visceral fat accumulation. Asian populations have higher abdominal fat than Europeans [12], and Asian often exhibit a unique body composition known as “thin-fat obesity”, characterized by increased visceral fat despite low BMI [27]. Since several obesity indices were developed and validated using data principally from White populations, their applicability to Asian population may be limited. Both the Chinese-derived CVAI and the Korean-derived NVAI are based on variables associated with abdominal visceral adiposity, such as WC, TG, and HDL-cholesterol, but they differ in their additional components: CVAI incorporates age and BMI, whereas NVAI includes age and mean blood pressure [28, 29]. In this study, we evaluated the associations between obesity and CKD in patients with DM using four anthropometric-based indices and four combined anthropometric–biochemical indices. Traditional anthropometric-based obesity indices, such as BMI and WHtR, may be less accurate in reflecting visceral fat burden and metabolic risk [30]. The inclusion of biochemical parameters, such as serum TG and HDL-cholesterol, provides a more comprehensive assessment of obesity-related risk than traditional anthropometric markers, because these indices capture not only the physical accumulation of adipose tissue but also its metabolic dysfunction [11]. By incorporating lipid profiles (TG and HDL), biochemical–anthropometric indices may better distinguish pro-inflammatory visceral fat from subcutaneous fat [31]. In addition, reductions in insulin sensitivity are driven not only by increased visceral fat mass but also by metabolic disturbances reflected in triglyceride and HDL cholesterol levels and an altered visceral-to-subcutaneous adipose tissue ratio [32]. This phenotype is associated with leptin resistance, systemic insulin resistance, lipotoxic cardiomyopathy, and endothelial dysfunction [33]. Accordingly, obesity indices incorporating biochemical parameters may provide greater clinical relevance for CKD risk assessment in diabetic populations. The risk of CKD in patients with DM increased with higher BMI, WHtR, BRI, CI, LAP, VAI, CVAI, and NVAI after adjusting for relevant covariates (all *P* for trend < 0.05) in this study. Although the effect sizes were broadly comparable between anthropometric-based and anthropometric–biochemical indices, NVAI demonstrated the strongest association with CKD risk in patients with DM (OR 2.866). When analyzed by sex, the association remained significant in males, whereas it showed borderline significance in females, with NVAI demonstrating the highest OR among all tested indices (*P* = 0.07).

Despite the overall associations between obesity-related indices and CKD in patients with DM, the strength and significance of these relationships differed by sex. Our study revealed distinct sex-specific patterns: all obesity-related indices were positively associated with CKD in males, whereas only LAP, VAI and CVAI were significant in females. The reasons for these sex differences remain unclear but may be related to sex differences in fat distribution. Females generally have a higher proportion of subcutaneous fat than males; therefore, WC may be less reflective of visceral fat accumulation in females [34]. Consistent with our findings, Yamasaki et al. reported that BMI, WC, and WtHR were significantly associated with CKD in males, whereas these associations were weaker in females, suggesting sex-specific differences in their utility of anthropometric indices as CKD predictors [35]. In addition, several studies have demonstrated that obesity markers are associated with an increased risk of kidney function decline in males, but not in females [31, 36]. Beyond differences in regional fatty acid storage, sex-related variation in the regulation of regional fatty acid metabolism may further influence the metabolic and renal relevance of anthropometric indices in women [30]. The inclusion of biochemical parameters, such as serum TG and HDL-cholesterol, provides a more comprehensive assessment of obesity-related risk than traditional anthropometric markers, as these indices capture not only the physical accumulation of adipose tissue but also its metabolic dysfunction. By incorporating lipid profiles, biochemical–anthropometric indices may better distinguish pro-inflammatory visceral fat from subcutaneous fat [31]. In the present study, while LAP, VAI, and CVAI were significantly associated with CKD risk in females, NVAI demonstrated only borderline significance in DM patents (*P* = 0.07). Consistent with a previous study of the general Korean population [14], NVAI exhibited the highest OR in both males and females with DM. The lack of statistical significance of NVAI in females may be attributed to the limited sample size of this subgroup and may also be influenced by the protective effects of estrogen on the renal vasculature. To validate these findings and further elucidate the sex-specific impact of obesity indices on renal health, longitudinal studies with larger, more diverse cohorts are warranted.

This study has several limitations. First, the cross-sectional design of this study precludes causal inference between obesity-related indices and CKD in patients with DM. Consequently, the observed associations should not be interpreted as predictive or causal effects. Second, self-reported data and single-time-point labs may introduce bias and reduce accuracy. Third, the definition of CKD in this study eGFR ≤ 60 mL/min/1.73 m^2^ or dipstick proteinuria ≥ 1 + may have led to a conservative estimation of CKD prevalence, as it does not capture microalbuminuria, a critical early indicator of diabetic kidney disease [37]. Despite this potential for misclassification, this criterion has been extensively validated and adopted in large-scale epidemiological studies a, providing a pragmatic approach for population-based analyses where quantitative albuminuria data may be limited [38]. Fourth, despite statistical significance in our models, the clinical utility of these obesity-related indices in predicting CKD risk among patients with DM appears constrained by their modest effect sizes and discriminative performance. These findings suggest that while such indices provide insights into the metabolic burden of adiposity, they may offer limited discriminatory value when used as isolated surrogate markers. Consequently, our results highlight the necessity of integrating these indices into comprehensive, multifactorial risk assessment strategies rather than relying on them as primary diagnostic tools for CKD in clinical practice. Fifth, this study has certain limitations related to the available data in the KNHANES dataset. Detailed information on diabetes duration and comprehensive medication history was not fully accessible, which may result in residual confounding from unmeasured clinical factors. Additionally, because eGFR and proteinuria were integral components of the CKD definition, they were intentionally excluded as covariates in the multivariable analysis to avoid mathematical redundancy and over-adjustment. Consequently, while our results demonstrate a strong association between obesity indices and CKD, they should be interpreted as reflecting the overall clinical presence of the disease rather than an association strictly independent of baseline renal function. Finally, as the study was limited to a Korean population, generalizability to other ethnic groups may be restricted. Despite these limitations, the study has strengths, including a large, nationally representative dataset, statistical adjustments, and inclusion of both conventional and biochemical–anthropometric indices. By analyzing Asian-specific indices such as CVAI and NVAI and performing sex-specific analyses, the study identified indices most strongly associated with CKD, suggesting potential ethnic- and sex-specific associations that may guide tailored risk assessment in Asian populations.

In conclusion, this study demonstrated that several obesity-related indices were significantly associated with CKD in patients with DM. CKD itself is a heterogeneous condition driven by multiple factors; hence, a single obesity-related marker may not fully capture its risk profile. Notably, indices incorporating both anthropometric and biochemical parameters were more effective in identifying CKD, particularly in females.

## Data Availability

The datasets for this study can be found in the Korea Centers for Disease Control and Prevention database through the following URL: https://knhanes.kdca.go.kr/knhanes/sub03/sub03_02_05. Individuals, including international researchers who sign up for membership, can utilize raw data from this website. However, the data access process and user manual are written in Korean.
